# Severe pulmonic valve regurgitation due to histoplasma endocarditis

**DOI:** 10.1530/ERP-14-0103

**Published:** 2015-02-02

**Authors:** Ewa A Konik, Merri Bremer, Peter T Lin, Sorin V Pislaru

**Affiliations:** Division of Cardiovascular Diseases, Mayo Clinic, 200 First Street SW, Rochester, Minnesota, 55905, USA

## Abstract

A 67-year-old man with myelodysplastic syndrome, disseminated histoplasmosis, and mitral valve replacement presented with dyspnea and peripheral edema. Transthoracic echocardiography demonstrated abnormal pulmonic valve with possible vegetation. Color flow imaging showed laminar flow from main pulmonary artery into right ventricular outflow tract (RVOT) in diastole. The continuous wave Doppler signal showed dense diastolic envelope with steep deceleration slope. These findings were consistent with severe pulmonic valve regurgitation, possibly due to endocarditis. Transesophageal echocardiography demonstrated an echodense mass attached to the pulmonic valve. The mitral valve bioprosthesis appeared intact. Bacterial and fungal blood cultures were negative; however, serum histoplasma antigen was positive. At surgery, the valve appeared destroyed by vegetations. Gomori methenamine silver-stains showed invasive fungal hyphae and yeast consistent with a dimorphic fungus. Valve cultures grew one colony of filamentous fungus. Itraconazole was continued based on expert infectious diseases diagnosis. After surgery, dyspnea and ankle edema resolved. To the best of our knowledge, histoplasma endocarditis of pulmonic valve has not been previously reported. Isolated pulmonic valve endocarditis is rare, accounting for about 2% of infectious endocarditis (IE) cases. Fungi account for about 3% of cases of native valve endocarditis. Characterization of pulmonary valve requires thorough interrogation with 2D and Doppler echocardiography techniques. Parasternal RVOT view allowed visualization of the pulmonary valve and assessment of regurgitation severity. As an anterior structure, it may be difficult to image with transesophageal echocardiography. Mid-esophageal right ventricular inflow–outflow view clearly showed the pulmonary valve and vegetation.

**Learning points:**

- Identification and characterization of pulmonary valve abnormalities require thorough interrogation with 2D and Doppler echocardiography techniques.
- Isolated pulmonary valve IE is rare and requires high index of suspicion.
- *Histoplasma capsulatum* IE is rare and requires high index of suspicion.

## Background

The variability of clinical presentation of infectious endocarditis (IE) requires high index of suspicion to make correct diagnosis. Echocardiography is central to the diagnosis and management of patients with IE. Isolated native pulmonic valve IE is rare, accounting for up to 2% of IE cases. Fungi account for up to 3% of cases of native valve IE. Patients who develop fungal endocarditis usually have multiple predisposing conditions. Despite combined medical and surgical interventions, mortality rates for fungal IE are high (1, 2). *Histoplasma capsulatum* is rare but important cause of fungal IE. Histoplasmosis is a common mycosis in endemic areas, particularly in North and Central Americas. It is usually asymptomatic, but can result in severe illness with protean manifestations.

## Case presentation

A 67-year-old man residing in Midwest USA presented with dyspnea on exertion, peripheral edema, and tachycardia (5/2014). His symptoms developed over a course of a few weeks.

**Figure 1:**
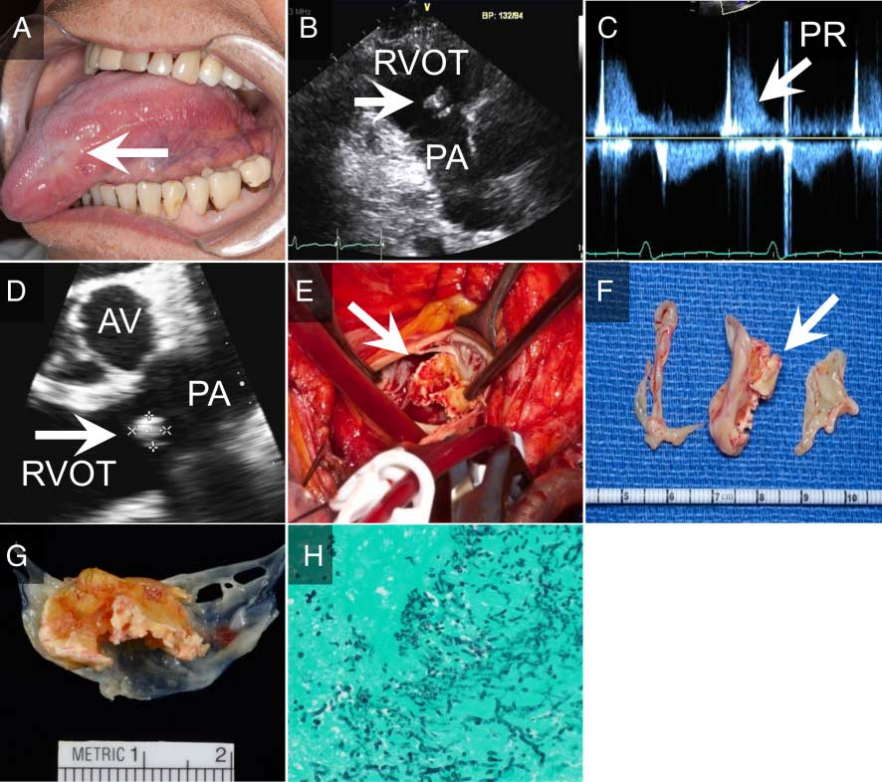
(A) Tongue lesion – the initial presentation of disseminated histoplamosis; (B) transthoracic echocardiogram: abnormal pulmonic valve with possible vegetation; (C) transthoracic echocardiogram: the continuous wave Doppler signal at the pulmonic valve showing dense diastolic envelope with a steep deceleration slope; (D) transesophageal echocardiogram: 1.4 cm×0.8 cm echodense mass attached to one of the cusps of the pulmonic valve; (E) intraoperative view of the pulmonic valve; (F) excised pulmonic valve; (G) one of the pulmonic valve cusps with attached vegetation; (H) gomori methenamine silver-stained sections of pulmonary valve showing invasive fungal hyphae and yeast.

Two years earlier (8/2012) he underwent mitral valve replacement for severe regurgitation due to bileaflet prolapse (33 mm Epic tissue valve). He had a long history of myelodysplastic syndrome and relapsing polychondritis, which was treated with prednisone and etanercept till 1 year before the presentation (5/2013) when he was diagnosed with disseminated histoplasmosis. The initial presentation was a tongue lesion and diagnosis was based on biopsy (Fig. 1A, arrow). During the same hospitalization, he underwent bronchoscopy for pulmonary infiltrates. BAL studies revealed *H. capsulatum* by fungal culture and pneumocystis carinii by PCR. He completed i.v. amphotericin course and has been treated with oral itraconazole 120 mg twice a day, maintaining therapeutic blood levels. Histoplasma urine antigen was not quantifiable, and thus not used for serial evaluation of therapeutic effect. Plasma histoplasma antigen was not measured. Patient has been treated with sulfamethoxazole–trimethoprim 160–800 mg four times a week as a chronic suppressive therapy for pneumocystis carinii pneumonia. Etanercept was switched to hydroxychloroquine 400 mg once a day by oral administration for symptomatic relapsing polychondritis, and prednisone was tapered down.

## Investigation

Physical examination revealed blood pressure of 120/80 mmHg with regular heart rate of 105 beats/min. There was mild lower extremity edema, no jugular vein distension, lungs clear to auscultation. There was a 3/6 mid systolic murmur at the mid left sternal border, with a faint diastolic murmur. S2 was persistently split. Blood sample analysis reported hemoglobin of 11.5 g/dl, mean corpuscular volume (MCV) 117 fl, leukocytes 5.9×10^9^/l, platelets 128×10^9^/l. Erythrocyte sedimentation rate (ESR) was 19 and C-reactive protein (CRP) was 6.7 mg/l (normal <8). Electrolyte panel and renal function were normal. Electrocardiogram (ECG) showed sinus tachycardia, 108 beats/min. Chest X-ray revealed mild fibrotic opacities in the lung bases and status post sternotomy.

Given the symptoms and signs of heart failure, the transthoracic echocardiography was performed. It demonstrated abnormal pulmonic valve with possible vegetation (Fig. 1B, arrow). Color flow imaging showed laminar flow from main pulmonary artery (PA) into right ventricular outflow tract (RVOT) in diastole (Video 1). The continuous wave Doppler signal at the pulmonic valve showed dense diastolic envelope with steep deceleration slope (Fig. 1C). The right ventricle was mildly enlarged with moderately decreased systolic function. These findings were consistent with severe pulmonic valve regurgitation, possibly due to endocarditis.

Video 1 Transthoracic echocardiogram: color flow imaging showing laminar flow from main pulmonary artery (PA) into right ventricular outflow tract (RVOT) in diastole. Download Video 1 via http://dx.doi.org/10.1530/ERP-14-0103-v1.
Download Video 1

To further investigate the pulmonic valve and the mitral valve bioprosthesis, the transesophageal echocardiography was performed. It demonstrated a 1.4 cm×0.8 cm echodense mass attached to one of the cusps of the pulmonic valve and prolapsing into the RVOT and PA (Fig. 1D). The mitral valve bioprosthesis appeared intact.

Pulmonic valve was imaged with transthoracic echocardiography in May 2013 (1 year before current evaluation) when the patient was diagnosed with disseminated histoplasmosis. It revealed thickened pulmonic valve with mild regurgitation (eccentric jet). The right ventricle was borderline enlarged with probably normal systolic function. Right ventricular systolic pressure was estimated at 41 mmHg.

Blood cultures for bacteria, fungi, and mycobacteria, as well as workup for culture-negative endocarditis were negative. Urine histoplasma antigen was negative. However, serum histoplasma antigen was positive (2.7 ng/ml).

## Treatment and outcome

Patient was evaluated by the infectious disease specialist and cardiac surgeon. Given high suspicion of IE, he was empirically treated with ceftriaxone 2 g once a day intravenously for 4 weeks followed by surgery.

Pulmonic valve was replaced with Hancock II 25 mm porcine bioprosthesis and tricuspid valve was repaired with annuloplasty ring. Excised pulmonary valve demonstrated thickened and retracted cusps, with one cusp completely destroyed with multiple, old vegetations (Fig. 1E, F, and G). The surgical specimen was sent for microbiologic and pathologic evaluation.

Gross examination of the pathologic specimen demonstrated large infective vegetation. Gomori methenamine silver-stained sections of pulmonary valve showed invasive fungal hyphae and yeast consistent with a dimorphic fungus (Fig. 1H). Fungal smear from excised material showed hyphae. The valve cultures grew one colony of filamentous fungus. The microscopic fungal species identification was impossible as the fungus appeared as sterile hyphae without micro- or macroconidia. DNA sequencing results were compatible with multiple fungal species, thus genus and species identification was impossible. Given prior history of disseminated histoplasmosis (based on fungal cultures from bronchoalveolar lavage fluid), detection of serum histoplasma antigen, the diagnosis of histoplasma endocarditis was made by infectious disease experts. Long-term itraconazole treatment continued. Patient is currently 7 months post-surgery and remains asymptomatic. Transthoracic echocardiogram performed at his last visit in January 2015 revealed normally functioning pulmonary valve prosthesis with mean gradient of 10 mmHg and no prosthetic or periprosthetic regurgitation. Most recent test for histoplasma antigen in serum was negative (value 0 ng/ml) on October the 10th, 2014. The next check up is planned for February 2015.

## Discussion

Identification and characterization of pulmonary valve abnormalities require thorough interrogation with 2D and Doppler echocardiography techniques. However, these abnormalities in adult patients may be more difficult to visualize with transthoracic echocardiography due to interference by underlying lung. Here, use of the transthoracic parasternal RVOT view allowed clear visualization of the pulmonary valve and vegetation, and facilitated assessment of pulmonary regurgitation severity. As an anterior, far-field structure, the pulmonary valve may also be difficult to image with transesophageal echocardiography. In this case, the pulmonary valve and vegetation were clearly visualized from the mid-esophageal right ventricular inflow–outflow view.

Isolated native pulmonic valve endocarditis is a rare entity, accounting for up to 2% of all IE cases. Infection due to fungal species occurs in ∼1–3% of all instances of native valve endocarditis and 5–7% of patients with prosthetic valve endocarditis. A review of fungal IE cases reported during 1965–1995 (3) and 1995–2000 (4) identified *H. capsulatum* as the etiologic agent in 15 of 270 (6%) and two of 152 (1%) cases respectively. The largest series on *H. capsulatum* IE encompasses 43 cases published between 1943 and 2003 (5). Fourteen more cases of *H. capsulatum* IE were reported during 2003–2012, at medical centers throughout the USA (6). To the best of our knowledge, isolated Histoplasma endocarditis of pulmonic valve has not been previously reported.

In addition to the rarity of the disease, there are two other unique features of the presented case. One is the fact that the patient presented 1 year after completing i.v. amphotericin treatment course and while on chronic itraconazole therapy for disseminated histoplamosis. This fact makes it challenging to compare the yield of our diagnostic work up with that in published reports. The other unique feature is the mitral valve bioprosthesis that was implanted 1 year before the diagnosis of disseminated histoplasmosis remained intact. One would expect that a bioprosthetic valve on the left side of the heart would be at greatest risk for infection. Possible predisposing factors for pulmonic valve involvement include history of relapsing polychondritis. Cardiovascular system, including cardiac valves, is involved in relapsing polychondritis (7). In addition, the patient had history of recurrent ear abscesses, treated with cephalexin, which might have possibly might have caused right-sided valves damage from bacterial seeding. Immunosuppression (etanercept, prednisone) and myelodysplastic syndrome might have predisposed the patient to fungal infection in this unusual location.
